# Aluminium as a Base for Artificial Teeth

**Published:** 1866-09

**Authors:** N. C. Keep

**Affiliations:** President, and Communicated for the Boston Medical and Surgical Journal


### ALUMINIUM AS A BASE FOR ARTIFICIAL TEETH.

(Read before the Massachusetts Dental Society, Jan. 8th, 1866, by N. C. Keep, M.D., President, and commnnicated for the Boston Medical and Surgical Journal.

Discovered by Wöhler in 1828, aluminium was known only by name until the genius of Deville, aided by the patronage of the French Emperor, overcame the obstacles which up to that time had proved insurmountable, and presented to the world the pure metal in a form available for use. At first it was produced in small quantities and at very high cost. I well remember the first specimen which came across the Atlantic, which cost more than its weight in gold.

The announcement of a new metal available for practical purposes made a great sensation throughout the civilized world. Every cabinet wanted a specimen, and for a long time the whole production was absorbed for this purpose. Still there were forcastings as to the uses to which this metal might be applied, when by improved processes it should become abundant and comparatively cheap. I well remember my own high hopes that I might use it instead of gold, and my disappointment when I found I did not possess the knowledge of the peculiar laws by which it could be made into plates, &c., and after a moderate effort I laid aside my specimens.

So far as I know, Dr. C. A. Fowler, of our own State, has the enviable distinction of being the first by his perseverance to actually overcome these difficulties, and to him I am indebted for the metal in a ductile form and the plan of using it without solder. In his process all the different parts are united by rubber, which has first been combined with comminuted aluminium. With this material all vacancies are obviated, desirable forms made, and the unity of the piece secured. When vulcanized and polished, it has a metallic lustre and an approximate resemblance to the metallic plate. The use of aluminium by jewellers, and their appreciation of its value, are sufficiently well known.

The advantages of alluminium in dentistry are:—

1st. It is sufficiently strong and unyielding (having almost no elasticity) to meet all the forces which it may be legitimately called upon to resist.

2d. Its very low specific gravity, 2-40, only 2½ half times heavier than water, enables the dentist to offer to his invalid patient a lighter set of teeth than by any other material now in use for base.

3d. Aluminium is not discolored by sulphuretted hydrogen, and is not acted upon by any acids which are likely to be found in the mouth, or by any of the secretions of the mouth, and does not discolor in use.

4th. Alluminium is a pure metal. All alloys are to some extent subject to galvanic action. It is well known that while gold plates were generally used, intelligent and conscientious dentists made their gold plates not less than three-fourths pure gold and one fourth alloy, and would have used pure gold had it not been too soft for plates. Ignorant and unscrupulous dentists often used a much larger per cent. of alloy, to the great discomfort of the wearer. Aluminium for plates should be pure; there is no excuse for introducing any alloy. They will thus be entirely free from the galvanic action incident to alloys.

5th. Aluminium is entirely innocuous. Neither the medal nor its salts can become poisonous. Plates of aluminium are easily brushed and kept free from foreign accumulations.

6th. They have the advantage over rubber in being strong and thin, taking up very little room in the mouth. Being thin, they do not give the impression of heat, which troubles some persons very much.

7th. By using aluminium we avoid the bi-sulphuret of mercury, which, though a soluble article, has excited solicitude in many minds; and as it constitutes about one half of the "red rubber" we would shun even the appearance of evil and let it alone.

In conclusion, we may congratulate the public that a new article is presented for their choice in artificial dentisty that fills more of the conditions desirable than any other at present or heretofore in use; and we confidently expect that those who have suffered from the weight of their artificial teeth, or from the galvanic action of mixed metals, or the yielding of elastic materials, or from uncomfortable thickness of rubber, or from the fear of injury by reason of the poisonous coloring matter used in red rubber, or from the sensation of heat occasioned by the rubber, which as now used is a bad conductor of caloric, or those who may desire a more artistic, highly-finished, easily cleansed, agreeable and comfortable substitute, will have occassion to thank M. Deville for producing alluminium in available quantity, and Dr. Fowler for his successful perseverance in bringing it into practical use for dental purposes.

To the dentist who should ask whether he had better abandon all other articles and attach himself to this only as a base for artificial teeth, I would say, this is not adapted to cheap dentistry. In the first place, the use of aluminium for dental purposes is patented, and we have no reason to think this patent "unjust or illegal." Then the labor and care in working being much greater than is now generally bestowed on rubber work, if you are contented and your patients are satisfied, these improvements will do you no good. But to the lover of progress, aiming at perfection, and to those who do not shrink from labor for the "substitutes" which can be produced, I cordially recommend a trial.

